# Closing the Gap: Investigation of Various Approaches in the Management of Patent Ductus Arteriosus

**DOI:** 10.7759/cureus.45009

**Published:** 2023-09-11

**Authors:** Farhana Ghouse, Claudia Idrobo Zapata, Pavan K Kasam Shiva, Anne Aguilar, Rithika Siripragada, Nandini Nair, Emiliano Vera, Amrita Suresh

**Affiliations:** 1 Medicine, Saint James School of Medicine Saint Vincent, Arnos Vale, VCT; 2 Pediatrics, Pontificia Universidad Javeriana, Bogotá, COL; 3 Internal Medicine, Bangalore Medical College and Research Institute, Bangalore, IND; 4 Internal Medicine, Universidad Popular Autónoma del Estado de Puebla, Puebla, MEX; 5 Internal Medicine, Sri Ramachandra Institute of Higher Education and Research, Chennai, IND; 6 Pediatrics, Byramjee Jeejeebhoy Government Medical College, Pune, IND; 7 Internal Medicine, Universidad Autónoma de Guadalajara, Guadalajara, MEX; 8 Internal Medicine, Kasturba Medical College, Mangalore, IND

**Keywords:** transcatheter closure, ibuprofen, indomethacin, pda, patent ductus arteriosus

## Abstract

In preterm newborns with extremely low birth weights, patent ductus arteriosus (PDA), which is defined as a remnant connection between the aorta and pulmonary artery after 72 hours of birth, is frequently linked to substantial morbidity and mortality. If left untreated, a hemodynamically significant PDA (hsPDA) increases the risk for bronchopulmonary dysplasia, necrotizing enterocolitis, and intraventricular hemorrhage among other morbidities, and can even lead to death. While instances of patent ductus arteriosus (PDA) resolving on their own are frequent, the primary approach for managing PDA closure in premature infants involves pharmacological interventions, commonly utilizing indomethacin, ibuprofen, or paracetamol. However, with these pharmacological treatment options, there is an increased risk of renal toxicity, gastrointestinal bleeding, and reopening of PDA among other complications. If pharmacological interventions are not successful or contraindicated, PDA can be closed via transcatheter closure or surgical ligation. As with any medically invasive procedure, it is not without risks and can lead to long-term complications. This review explores the different management options and the benefits and outcomes of conservative management vs. active management in order to get one step closer to standardizing the treatment for PDA. With so much controversy surrounding the best management option, there is a lack of evidence to support one treatment method superior to the other in reducing overall mortality, and this needs to be explored further.

## Introduction and background

Patent ductus arteriosus (PDA) constitutes 5-10% of congenital heart issues in full-term newborns and is even more common in preterm infants with low birth weights [1,2]. During fetal circulation, the ductus arteriosus plays a vital role in directing blood flow from the fetal pulmonary artery to the aorta, effectively bypassing the underdeveloped lungs [3]. At birth, this anatomical connection constricts and forms a fibrous tissue, ligamentum arteriosum, due to increased arterial oxygen content, decreased pulmonary vascular resistance, and decreased prostaglandin E2 levels [3]. If the ductus arteriosus fails to close 72 hours after birth, it is defined as PDA. In preterm infants <28 weeks and low birth weights <1500 g, the risk of failure of ductus arteriosus to close is greater than 50%, with the birth weight and incidence of PDA being inversely proportional [4,5]. When hemodynamically significant, PDAs have been associated with morbidity and mortality of up to 30% [1]. Complications of PDA can include pulmonary hyperperfusion and systemic hypoperfusion [6]. Clinically, this manifests as pulmonary congestion, edema, dyspnea, bronchopulmonary dysplasia, necrotizing enterocolitis, intraventricular hemorrhage, periventricular leukomalacia, and renal ischemia [6].

PDA is classically managed pharmacologically with ibuprofen, indomethacin, and acetaminophen [7]. If pharmacological treatments fail, interventional procedures are usually performed to close a PDA. Even though indomethacin and other pharmacological options have been the predominant method of management, upcoming evidence has favored expectant management over pharmacological and surgical options. Clinical studies have recently supported that expectant management is non-inferior to pharmacological management and can be equally effective in the closure of PDA while avoiding the risk of serious side effects [8]. This narrative review aimed to provide a comprehensive overview of PDA and discuss the different approaches and interventions available for managing PDA. Additionally, given the conflicting strategies for treating PDA, this review will assess the effectiveness and safety of different management approaches, identify controversies and challenges in PDA management, and make recommendations for future research direction.

## Review

Overview of management

Over the years, the method and timeline of PDA management have been constantly debated. Conventionally, non-steroidal anti-inflammatory drugs, specifically indomethacin and ibuprofen, along with acetaminophen, are used to close PDAs [8]. Surgical interventions used prophylactically or in the event of failure of PDA closure with pharmacological agents include transcatheter closure or surgical ligation [9]. However, recent studies have been investigating conservative management including fluid restriction, diuretics, and ventilatory adjustments as management options.

A study by Mitra et al. concluded that when 4256 infants were treated pharmacologically with 14 different variations of indomethacin, ibuprofen, or acetaminophen, the overall rate of PDA closure was 67.4% [8]. Although pharmacological management is widely used for the closure of PDAs, the most effective route and dosage of medication are still being explored. Administering a higher dose of oral ibuprofen resulted in a significantly increased rate of closure of patent ductus arteriosus compared to the standard intravenous dosage (OR: 3.59, 95% CI: 1.64-8.17 vs. OR: 2.35, 95% CI: 1.08-5.31) [8].

Furthermore, the risks and benefits of pharmacological treatments in preterm infants are still being investigated. While indomethacin is an effective method of management, a study explored the effect of indomethacin therapy on renal function in 11 preterm infants with very low birth weight presenting with hemodynamically significant PDA (hsPDA), which demonstrated a decrease in diuresis and creatinine clearance as well as an increase in body weight [10]. Furthermore, new evidence suggests that acetaminophen is as effective as ibuprofen, but with fewer adverse effects in the closure of PDA, and no significant difference was found when comparing the two drugs [11].

A randomized controlled trial by Potsiurko et al. compared pharmacological treatment within 72 hours of conservative management [12]. They concluded that early pharmacological intervention provided more frequent and earlier closure of PDA in preterm infants, but it did not decrease morbidity nor improve survival rates [12]. Additionally, Hundscheid et al. showed non-inferiority when comparing expectant management to early ibuprofen in overall mortality (14.0% vs. 18.2%) [13].

Mosalli and Alfaleh investigated the effect of prophylactic surgical ligation in PDA and concluded that in infants <28 weeks with birth weight <1000 g, a significant reduction of necrotizing enterocolitis was observed [14]. However, no mortality benefit was observed [14]. Nemerofsky et al. reported that 94% of infants with birthweight >1000 g did not require intervention and the PDA closed spontaneously [15]. They also concluded that deferring treatment decisions in infants with birth weight <1000 g, until they have reached at least one week of age could potentially prevent unnecessary exposure to different treatment options [15].

According to a study by Gillam-Krakauer and Reese, infants with few risk factors and birth weights >1000 g can generally be conservatively managed, whereas infants with birth weights of <1000 g and more risk factors should be conservatively managed before starting pharmacological treatment if there is no resolution by the second week of life or if morbidities are present [16]. A recent meta-analysis by Cheema et al. found no difference between expectant management and pharmacological management of PDA regarding mortality and other adverse clinical outcomes [17].

Although some studies have shown a reduction in morbidity with various treatment modalities, the long-term effects of conservative vs. active management on mortality prove to be challenging and need to be investigated further. The risks and benefits of earlier vs. later treatment timelines and the adverse effects of pharmacological and surgical options compared to conservative management also need to be explored further.

Conservative management

Conservative management for PDA pertains to interventions including fluid restriction, diuretics, or ventilator adjustments [18]. Fluid restriction is commonly practiced in preterm infants with PDAs, particularly in the setting of enteral feedings due to the increased risk of necrotizing enterocolitis [19]. A study by Stephens et al. suggested that limiting fluid intake during the first three days of life is protective of PDA [20]. Further investigations revealed a threshold of fluid administration, when surpassed increased the risk of PDA significantly. The threshold per day was found to be 146 mL/kg on day one and over 170 mL/kg during the rest of the first week of life and caloric intake seemed to be unaffected [20]. A study conducted by De Buyst et al. revealed that reduced fluid intake in the initial days of preterm infants' lives was linked to a lower likelihood of developing bronchopulmonary dysplasia and PDA [21]. It is important to note that this decrease in blood volume may have potentially made significant alterations in neuroendocrine mechanisms [21]. Therefore, the risks and benefits of fluid restriction need to be investigated further. 

An elevation in postnatal oxygen levels has a significant impact on the initial constriction of the ductus arteriosus, leading to its eventual closure [22]. Noori et al. conducted a study and discovered that maintaining arterial oxygen saturation in the range of 83-89% increased the occurrence of patent ductus arteriosus, but did not affect the need for surgical ligation of the duct [22]. These findings indicate that the lower saturation range applied in the study might coincide with the minimum PaO_2_ threshold required for effective constriction of the DA; however, further research is needed to determine what minimum PaO_2_ level is required specifically to trigger the initial constructive response. Additionally, pharmacological interventions designed to induce closure of PDA were found to be equally effective when administered with an oxygen saturation ranging from 83% to 89% [22].

In premature infants with PDA, furosemide, a diuretic, is typically used to avoid adverse pulmonary and cardiovascular effects seen with fluid overload [23]. However, a study by Green et al. concluded that in premature infants with respiratory distress syndrome, furosemide increased the occurrence of PDA due to increased production of prostaglandin E2 [23]. A review by Brion and Campbell found that after giving furosemide in addition to indomethacin, a significant increase in the risk of failure of ductal closure was not seen; however, a larger sample size would be needed to confirm this finding [24].

Hundscheid et al. found that when comparing expectant management to early ibuprofen treatment in extremely premature infants from postnatal age of 24 to 72 hours, the former showed non-inferiority in regards to necrotizing enterocolitis (17.6% vs. 15.3%), (RD: 2.3, 95% CI: -6.5 to 11.1), bronchopulmonary dysplasia (33.3% vs. 50.9%), (RD: -17.6, 95% CI, -30.2 to -5.0) or death (14.0% vs. 18.2%), (RD: -4.3, 95% CI: -13.0 to 4.4) [13]. This conclusion reinforces the previous evidence regarding the lack of beneficial outcomes of pharmacological PDA treatment on clinical outcomes.

Furthermore, a meta-analysis of four randomized controlled trials revealed no differences in mortality or morbidity, (RR: 1.09 {0.73-1.61}) when comparing conservative management with pharmacological and surgical treatment options [18].

Pharmacological treatment

Indomethacin has been used traditionally for the non-surgical closure of PDA. This medication effectively inhibits the synthesis of prostaglandins, which play a crucial role in maintaining the patency of the ductus arteriosus [25]. A national collaborative study done in 1983 included 405 infants, 79% of them who were given indomethacin had hemodynamical closure of the PDA in the first 48 hours [26]. Among those, the reopening rate was 26% without needing surgical correction further in the trial. However, short-term renal toxicity was found to be associated with the use of indomethacin [26]. A review article including more than 77 published articles concluded that indomethacin when given in infants between 23 and 24 weeks of life for the prevention of PDA, decreased its incidence by 73% [27].

Even though the exact mechanism of action of ibuprofen remains unknown, it is believed to be related to the inhibition of prostaglandin synthesis via the inhibition of cyclo-oxygenase enzyme-1 and -2 [28]. A systematic review of various studies done in Toronto that included 24 studies to compare ibuprofen vs. indomethacin concluded that there was no statistically significant difference in failure of closure rates of PDA (RR: 1.07, 95% CI: 0.92-1.24) between the two groups [29]. Nephrotoxicity due to the use of ibuprofen; however, remained a concern among premature neonates in NICU [30].

Researchers have recently taken a keen interest in exploring the utilization of acetaminophen to close patent ductus arteriosus (PDA) [31]. Acetaminophen is an inhibitor of the prostaglandin synthase enzyme’s peroxidase component, decreasing the synthesis of PGs that leads to the closure of PDA [30]. A review article by Bardanzellu et al. found that paracetamol use had a safer profile compared to ibuprofen use with similar efficacy in PDA closure with p-value [30]. However, an increased risk of intestinal bleeding was seen to be associated with paracetamol use [30].

A systemic review and meta-analysis evaluating the role of different pharmacological treatment options in PDA management used data from two RCTs and 14 uncontrolled trials [31]. The two RCTs found no significant difference between acetaminophen and ibuprofen regarding PDA closure (RR: 1.07; 95% CI: 0.87-1.33) [31]. The study concluded that among the uncontrolled studies done, there was a significant improvement in the subjects when acetaminophen was used as first-line therapy for children with gestational age >28 days (RR: 1.03; 95% CI: 0.92-1.16). It also concluded that the preferred route of administration is oral at lower doses [31].

Another meta-analysis of 70 studies evaluated the efficacy and safety of various treatment options for PDA closure [32]. They found that high-dose oral ibuprofen is the best pharmacological treatment option for PDA closure in preterm and term infants with hsPDA [32]. These findings were further replicated by another systematic review, which found that high-dose oral ibuprofen has greater efficacy in patients with hsPDA as compared to standard-dose ibuprofen or IV indomethacin [8].

Interventional procedures 

In situations where pharmacological treatment using non-selective COX inhibitors proves ineffective or contraindicated, or when spontaneous closure is unsuccessful, interventional procedures become warranted for the management of PDA. These interventional procedures include transcatheter closure or surgical ligation [9]. Transcatheter closure is maintained as the standard of care in children and infants weighing >6 kg. However, outcomes regarding the transcatheter approach in low body weight and preterm infants remain inconclusive [33].

A multicenter, retrospective study performed by Dimas et al. analyzed the outcome of transcatheter closure of PDA in infants weighing ≤6 kg [34]. Complete occlusion was observed in 94% of neonates which concluded transcatheter PDA closure to be efficacious and safe in infants weighing ≤6 kg. However, data regarding outcomes of transcatheter PDA closure in very preterm infants remains inconclusive. Another study concluded that transcatheter coil occlusion is efficacious in closing PDA (87.5%) in symptomatic preterm infants and can be used as an alternative to surgical ligation [35].

Paudel et al. evaluated the use of transthoracic echocardiography (TTE) during transcatheter closure of PDA in extremely low birth weight infants [36]. TTE was found to be the preferred technique for choosing the appropriate device size compared to conventional angiograms as aortic angiograms require femoral artery access which is associated with risks such as sudden loss of pulse [37].

Surgical intervention can be done by a postero-lateral thoracotomy or by the recently advancing video-assisted thoracoscopic surgery (VATS). Although no major differences in postoperative complications and clinical outcomes are reported, VATS has been observed to be a more cost-effective procedure with a shorter hospital stay and rapid recovery time [38]. In a comparative study conducted by Mandhan et al., the effectiveness of two different techniques (suture ligation and clip application) for surgical ligation was investigated [39]. The study, which included 67 preterm neonates, revealed that postoperative complications occurred in six infants from the suture ligation group, while only two infants from the clip application group experienced complications. Clip application was found to be superior to suture ligation due to its associated benefits such as reduced operative time and morbidity [39].

The study conducted by Lehenbauer et al. analyzed the effects of surgical ligation on 166 preterm neonates with PDA [40]. The findings indicated that while a small percentage (2.4%) experienced postoperative complications, a significant majority (54.1%) were relieved from inotropic support within the first day following surgery. Furthermore, the observed 30-day mortality rate was low at only 1.8%. Based on these results, it can be concluded that surgical ligation is a safe and effective treatment option for managing PDA in preterm neonates [40]. Long-term complications of these interventional procedures include left vocal cord paralysis (52%), diaphragmatic paralysis (3.4%), scoliosis, and rarely death [41,42].

Controversies and challenges in PDA management

Controversy continues regarding the appropriate time of treatment of the PDA due to the lack of a standardized approach for all preterm infants [43,44]. In 1982, Mahony et al. found that low birth weight (<1000 g) infants at birth are at a high risk of hemodynamically unstable PDA, and early treatment with indomethacin decreases morbidity without increasing complications [45]. More recently, Sosenko et al. found no difference in respiratory outcomes (OR: 0.17, 0.03-0.88), death (OR: 0.27, 0.07-0.99), and other complications of prematurity after administering early ibuprofen at the onset of subtle patent PDA symptoms compared with expectant ibuprofen management only when the condition becomes hemodynamically significant [46].

Gudmundsdottir et al. reviewed whether either early, intermediate, or late pharmacological management is related to adverse outcomes in extremely preterm infants [47]. Compared to early therapy, they showed no increase in the risk of secondary invasive PDA closure, or mortality after an intermediate or a late intervention ({aHR: 0.89, 95% CI: 0.57-1.39} and {aHR: 1.10, 95% CI: 0.53-2.28} respectively). Regarding the incidence of chronic pulmonary disease, the intermediate therapy was not associated with any risk (aOR: 0.83, 95% CI: 0.42-1.64), and a late intervention was associated with a lower risk (aOR: 0.28, 95% CI: 0.13-0.61) [47].

Prophylactic management of asymptomatic PDA is another area of debate. Fowlie et al. showed no difference in mortality or long-term neurosensory impairment between prophylactic indomethacin vs. placebo (RR: 1.02, 95% CI: 0.90-1.15) [48]. However, the incidence of symptomatic PDA and intraventricular hemorrhage (IVH) was significantly decreased with the administration of prophylactic indomethacin ({RR: 0.44; 95% CI: 0.38-0.50} and {RR: 0.66, 95% CI: 0.53-0.82}). According to a Cochrane review, prophylactic indomethacin and ibuprofen have shown a decrease in the need for surgical closure of PDA ({RR: 0.51, 95% CI: 0.37-0.71} and {RR: 0.46, 95% CI: 0.22-0.96}, respectively) [49]. Prophylactic use of ibuprofen and indomethacin significantly reduced the incidence of IVH but prophylactic acetaminophen has an unclear effect on the incidence of IV [49]. In addition, there is no evidence regarding the effect of prophylactic interventions on important outcomes such as death, neurodevelopmental disability, or chronic lung disease [49].

However, the adverse effects of prophylactic therapy should be considered. Ibuprofen and indomethacin have been associated with a high risk of oliguria [29,48]. Prophylactic ibuprofen has been linked to an increased risk of gastrointestinal bleeding and pulmonary hypertension [49]. Indomethacin is associated with an increased incidence of spontaneous gastrointestinal perforation [49].

The American Academy of Pediatrics has made some recommendations to guide PDA treatment. Prophylactic therapy may be acceptable for infants less than 26 weeks’ gestation, and weight under 750 g in care units with low spontaneous closure rates [5]. Asymptomatic PDA treated with indomethacin seems to reduce the incidence of symptomatic PDA [49]. Therefore, extremely preterm infants less than 28 weeks gestation under six days of age who have moderate-severe hemodynamic shunt on echocardiography and require moderate respiratory support may be treated [5]. Similarly, pharmacologic therapy for symptomatic PDA may be considered. Invasive DAP closure is a reasonable alternative after a failed pharmacology intervention [5,49]. In summary, an individual approach regarding the benefits and harms of treatment due to the lack of clear evidence for benefit is recommended [5,49]. Further studies will be helpful in a better understanding of this condition and its management in order to improve preterm infants’ outcomes.

## Conclusions

Management of patent ductus arteriosus in neonates involves conservative, pharmacological, and surgical interventions. Over the past six decades, there have been significant technological advancements in PDA management. Interventional procedures such as transcatheter closure or surgical ligation may be necessary if pharmacological therapy fails or is contraindicated. However, it should be noted that surgical ligation has been associated with bronchopulmonary dysplasia while the use of indomethacin can lead to increased cases of interventricular hemorrhage and nephrotoxicity in newborns with PDAs. These adverse effects highlight the importance of considering conservative management as a viable alternative option for treating PDAs in neonates. In recent years, there has been a growing trend towards adopting conservative management approaches for this purpose. Conservative options like the use of diuretics, fluid restriction, and increasing oxygen saturation have been shown to be equally effective with reduced side effects according to data from the latest studies.

To compare the efficacy of different treatment options, further large randomized trials need to be conducted and the results of studies with larger sample sizes and higher power can be used to draw better conclusions about safety, and long-term outcomes of management strategies. Clinicians must be aware of the implications of novel treatment modalities in PDA closure. The emergence of studies showing conservative management to be as efficient as active interventions is to be taken into consideration while managing neonates. PDA closure should be individualized catering to the needs of each neonate taking into account the short-term goals and long-term treatment outcomes and the effect on mortality and morbidity in patients.
