# Correction: Closing the Gap: Investigation of Various Approaches in the Management of Patent Ductus Arteriosus

**DOI:** 10.7759/cureus.c144

**Published:** 2023-11-17

**Authors:** Farhana Ghouse, Claudia Idrobo Zapata, Pavan K Kasam Shiva, Anne Aguilar, Rithika Siripragada, Nandini Nair, Emiliano Vera, Amrita Suresh

**Affiliations:** 1 Medicine, Saint James School of Medicine Saint Vincent, Arnos Vale, VCT; 2 Pediatrics, Pontificia Universidad Javeriana, Bogotá, COL; 3 Internal Medicine, Bangalore Medical College and Research Institute, Bangalore, IND; 4 Internal Medicine, Universidad Popular Autónoma del Estado de Puebla, Puebla, MEX; 5 Internal Medicine, Sri Ramachandra Institute of Higher Education and Research, Chennai, IND; 6 Pediatrics, Byramjee Jeejeebhoy Government Medical College, Pune, IND; 7 Internal Medicine, Universidad Autónoma de Guadalajara, Guadalajara, MEX; 8 Internal Medicine, Kasturba Medical College, Mangalore, IND

The affiliation of Farhana Ghouse has been corrected at her request to change the location of Saint James School of Medicine from Park Ridge, United States to Arnos Vale, St. Vincent, St. Vincent and the Grenadines.

